# Albumin Adducts of Electrophilic Benzene Metabolites in Benzene-Exposed and Control Workers

**DOI:** 10.1289/ehp.8948

**Published:** 2006-09-25

**Authors:** Yu-Sheng Lin, Roel Vermeulen, Chin H. Tsai, Suramya Waidyanatha, Qing Lan, Nathaniel Rothman, Martyn T. Smith, Luoping Zhang, Min Shen, Guilan Li, Songnian Yin, Sungkyoon Kim, Stephen M. Rappaport

**Affiliations:** 1 Department of Environmental Sciences and Engineering, School of Public Health, University of North Carolina, Chapel Hill, North Carolina, USA; 2 National Cancer Institute, National Institutes of Health, Department of Health and Human Services, Bethesda, Maryland, USA; 3 School of Public Health, University of California, Berkeley, California, USA; 4 Chinese Center for Disease Control and Prevention, Beijing, China

**Keywords:** albumin adduct, benzene oxide, benzoquinone, nonlinear, variation

## Abstract

**Background:**

Metabolism of benzene produces reactive electrophiles, including benzene oxide (BO), 1,4-benzoquinone (1,4-BQ), and 1,2-benzoquinone (1,2-BQ), that are capable of reacting with blood proteins to produce adducts.

**Objectives:**

The main purpose of this study was to characterize relationships between levels of albumin adducts of these electrophiles in blood and the corresponding benzene exposures in benzene-exposed and control workers, after adjusting for important covariates. Because second blood samples were obtained from a subset of exposed workers, we also desired to estimate within-person and between-person variance components for the three adducts.

**Methods:**

We measured albumin adducts and benzene exposures in 250 benzene-exposed workers (exposure range, 0.26–54.5 ppm) and 140 control workers (exposure range < 0.01–0.53 ppm) from Tianjin, China. Separate multiple linear regression models were fitted to the logged adduct levels for workers exposed to benzene < 1 ppm and ≥ 1 ppm. Mixed-effects models were used to estimate within-person and between-person variance components of adduct levels.

**Results:**

We observed nonlinear (hockey-stick shaped) exposure–adduct relationships in log-scale, with inflection points between about 0.5 and 5 ppm. These inflection points represent air concentrations at which benzene contributed marginally to background adducts derived from smoking and from dietary and endogenous sources. Adduct levels were significantly affected by the blood-collection medium (serum or plasma containing either heparin or EDTA), smoking, age, and body mass index. When model predictions of adduct levels were plotted versus benzene exposure ≥ 1 ppm, we observed marked downward concavity, particularly for adducts of the benzoquinones. The between-person variance component of adduct levels increased in the order 1,2-BQ < 1,4-BQ < BO, whereas the within-person variance components of the three adducts followed the reverse order.

**Conclusions:**

Although albumin adducts of BO and the benzoquinones reflect exposures to benzene ≥ 1 ppm, they would not be useful biomarkers of exposure at ambient levels of benzene, which tend to be < 0.01 ppm, or in those working populations where exposures are consistently < 1 ppm. The concavity of exposure–adduct relationships is consistent with saturable metabolism of benzene at air concentrations > 1 ppm. The surprisingly large effect of the blood-collection medium on adduct levels, particularly those of the benzoquinones, should be further investigated.

Benzene is a ubiquitous environmental contaminant generated from petroleum products and from combustion of organic matter, including cigarette smoking. Air concentrations of benzene are typically < 0.01 ppm in ambient environments but can exceed 10 ppm in industrial settings where benzene-containing products are used [International Agency for Research on Cancer (IARC) 1989; Wallace 1996]. Workers exposed to benzene have consistently experienced increased risks of hematopoietic disorders and leukemias (Hayes et al. 2000; Lan et al. 2004; Savitz and Andrews 1997). These toxic effects are thought to result from metabolism of benzene to reactive products (Snyder 2002).

As shown in Figure 1, benzene metabolites include several reactive electrophiles, namely, benzene oxide (BO), 1,2- and 1,4-benzoquinone (1,2-BQ and 1,4-BQ, respectively), the muconaldehydes, and benzene diolepoxide [reviewed by (Snyder 2000a, 2000b, 2002)]. Because these electrophilic species are short lived *in vivo*, they have been investigated in animals and humans by measuring their adducts with hemoglobin, serum albumin, and bone-marrow proteins (Bechtold et al. 1992; McDonald et al. 1993; Rappaport et al. 2002a; Waidyanatha et al. 1998; Yeowell-O’Connell et al. 1996, 2001). Albumin adducts of BO (BO-Alb) and 1,4-BQ (1,4-BQ-Alb) accumulate over the course of 3–4 weeks in humans (Rappaport et al. 2002a) and thereby serve as intermediate-term biomarkers of exposure (Lin et al. 2005). Although albumin adducts of 1,2-BQ (1,2-BQ-Alb) have been reported in rats and mice to which benzene had been administered (McDonald et al. 1994; Troester et al. 2000; Waidyanatha et al. 1998), they have not been reported heretofore in humans.

In previous studies of benzene-exposed workers in China, nonlinear (concave-downward) exposure–adduct relationships were reported for BO-Alb and 1,4-BQ-Alb, and adduct levels were significantly affected by age and cigarette smoking (Rappaport et al. 2002a, 2005). However, those investigations did not explore exposure–adduct relationships among persons exposed to benzene in environmental air and cigarette smoke because of large background contributions of BO-Alb and 1,4-BQ-Alb from dietary and endogenous sources (Lin et al. 2006; McDonald et al. 2001). This raises questions regarding the range of benzene exposures over which BO-Alb and 1,4-BQ-Alb can serve as useful biomarkers.

In the present study, we report levels of BO-Alb, 1,4-BQ-Alb, and 1,2-BQ-Alb in 250 benzene-exposed workers and 140 control workers in Tianjin, China. Because individual benzene exposures were obtained for both exposed and control workers, we constructed exposure–adduct relationships over about 5 orders of magnitude of air concentrations and determined levels of exposure at which benzene-derived adducts can be differentiated from background adducts. We then identified effects on adduct levels of the blood-collection medium, age, body mass index (BMI), sex, and cigarette smoking. Because two blood samples were obtained from a subset of exposed workers, we also estimated within-person and between-person components of variance for the three albumin adducts. Finally, we considered the concavity of the exposure–adduct relationships for workers exposed to ≥ 1 ppm.

## Materials and Methods

### Recruitment of subjects

This study was approved by the Institutional Review Boards of the National Cancer Institute, the Chinese Academy of Preventive Medicine, the University of North Carolina at Chapel Hill, and the University of California, Berkeley. Exposed workers (*n* = 250) were recruited from two shoe manufacturing factories in Tianjin, China, and sex- and age-matched control workers (*n* = 140) were recruited from neighboring clothes manufacturing factories. Written informed consent was obtained at the time of enrollment, and a standardized questionnaire was administered to gather demographic and lifestyle information including medical history, current smoking status, and alcohol consumption (Lan et al. 2004).

### Exposure assessment

A detailed description of the exposure assessment was reported previously (Kim et al. 2006; Vermeulen et al. 2004). Briefly, occupational exposure to benzene, from the use of benzene-containing glues, was measured during each of 16 months over 2000–2001 with passive personal monitors (Organic Vapor Monitors; 3M, St. Paul, MN, USA) (Vermeulen et al. 2004). None of the workers used respirators. Because all benzene measurements among control workers were below the limit of detection (nominally 0.2 ppm), air levels were predicted for these subjects from postshift levels of urinary benzene (one to four measurements per person) (Kim et al. 2006). Air concentrations of benzene among exposed workers were estimated as geometric mean (GM) levels for all statistical analyses, using individual air measurements obtained within about 3 months of each blood sample (median: four measurements per person). The median interval between the first air measurement and blood collection was 90 days, and 90% of the intervals ranged between 54 and 103 days. For subjects with two blood specimens (*n* = 28), we used only data from the first sample for cross-sectional analyses.

### Measurement of albumin adducts

All subjects provided a single venous blood sample during either 2000 or 2001, and 28 exposed subjects provided blood specimens in both years. Eighty percent of the assays were conducted with serum, 12% with plasma containing heparin, and 8% with plasma containing EDTA. (Adducts were measured in plasma samples only in cases where serum was unavailable because of amounts of serum required for other assays.) Serum (or plasma) was separated from red cells immediately after phlebotomy and stored at −80°C until analysis. Samples were identified by randomly assigned numbers. Information about exposure levels and demographic factors were released after all assays had been completed and results had been shared with collaborators.

Albumin was isolated from serum or plasma, dried to constant weight, and analyzed by derivatization and gas chromatography-mass spectrometry, as described previously (Waidyanatha et al. 1998) with minor modifications. Briefly, to 5 mg albumin we added 5 μg [^2^H_4_]1,4-BQ-Alb, 10 μg [^2^H_4_]1,2-BQ-Alb, and 0.005 pmol [^2^H_5_]*S*-phenyl cysteine (internal standards). Samples were thoroughly dried and then reacted with tri-fluoroacetic anhydride and methanesulfonic acid to produce volatile fluorinated derivatives of the sulfur-bound adducts. Although the benzoquinones are capable of forming multi-*S*-substituted adducts and crosslinks, our assay only detected mono-*S*-substituted adducts. After concentrating the products under nitrogen, the residue was dissolved in hexane and then washed once with 0.1 M Tris buffer (pH 7.5) and twice with deionized water. The solution was concentrated under nitrogen to 200 μL and 1- or 2-μL aliquots were analyzed by gas chromatography-negative ion chemical ionization mass spectrometry in selected ion monitoring mode using an HP 5980 Series II-plus gas chromatograph, containing a DB-5 fused silica column (60 m, 0.25-mm i.d., 0.25-μM film thickness), and coupled to an HP 5989B MS engine (HP-Agilent, Santa Clara, CA, USA). We monitored the following ions: *m/z* 333 for 1,2-BQ-Alb and 1,4-BQ-Alb, *m/z* 336 for [^2^H_4_]1,2-BQ-Alb and [^2^H_4_]-1,4-BQ-Alb, *m/z* 206 for BO-Alb, and *m/z* 211 for [^2^H_5_]BO-Alb. Quantification was based on peak areas relative to the corresponding isotopically labeled internal standards.

### Statistical analyses

#### Summary statistics and assay precision

The precision of the adduct assays [expressed as a coefficient of variation (CV)] was determined from duplicate assays of 38 randomly selected albumin specimens that had been analyzed blind from the study population. The CV was estimated as


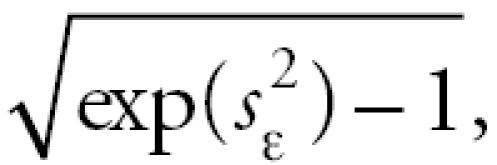


where *s*^2^_ɛ_ is the estimated error variance obtained from a one-way analysis of variance (ANOVA) of the log-transformed levels of each analyte. Chi-square statistics and ANOVA, or nonparametric Wilcoxon rank-sum tests (if the distributions were skewed), were used to examine the distributions of albumin adducts and demographic factors, stratified by exposure status.

#### Exposure–adduct relationships

Before building final regression models, non-parametric generalized additive models with loess smoothers (Hastie and Tibshirani 1999) were applied to explore nonlinear relationships between levels of albumin adducts and benzene exposures, after adjusting for covariates with the convergence criteria recommended by Dominici et al. (2002). Partial residual plots were also used to assess nonlinearity and to determine whether transformations of continuous covariates were needed. Because the distributions of adduct levels and benzene exposures were right-skewed and displayed nonuniform variances, we used natural logarithms of the observations for multiple linear regression models.

Segmented linear regression models were developed to investigate exposure–adduct relationships and covariate effects above and at or below a common exposure concentration for the three adducts. After considering various benzene concentrations between 0.1 and 3 ppm, we chose a value of 1 ppm because it maximized overall values of *R*^2^ and minimized overall values of the Akaike information criterion (AIC) for models of the three adducts. We used residual plots to assess the fits of final models. Candidate covariates were selected based upon preliminary univariate analyses and findings from previous studies. The generalized extreme-Studentized-deviation-many-outlier method (Rosner 1983) identified six outliers of 1,2-BQ-Alb among subjects exposed to < 1 ppm of benzene; these outliers were excluded from multiple regression models to ensure the accuracy and reliability of analyses.

#### Estimation of between-person and within-person variance components for levels of albumin adducts

Mixed-effects models, which account for the correlation between serial measurements on the same subjects, were used to estimate the between-person and within-person variance components for logged levels of BO-Alb, 1,2-BQ-Alb, or 1,4-BQ-Alb. Considering the blood-collection medium as a fixed effect, this model has the form


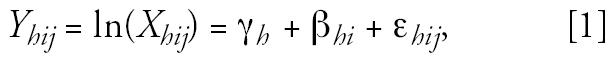


where *X_hij_* is the level of a specific albumin adduct for the *j*th observation (*j* = 1, 2) from the *i*th person (*i* = 1, …, 28) with the *h*th blood-collection medium (*h* = 1, 2, 3), *Y_hij_* is the natural logarithm of *X_hij_*, γ*_h_* is the fixed effect of the *h*th blood-collection medium, β*_hi_* is the random effect of the *i*th person with the *h*th blood-collection medium, and ɛ*_hij_* is the random-error effect of the *j*th observation from the *i*th person with the *h*th blood-collection medium. The random effects β*_hi_* and ɛ*_hij_* are assumed to be common to all blood-collection media, mutually independent, and normally distributed, with means of zero and variances of σ_B_^2^ and, σ_W_^2^ representing the between- and within-person variance components, respectively. We assumed a compound symmetric variance–covariance structure and used restricted maximum likelihood estimation. The estimates of σ_B_^2^ and σ_W_^2^ are designated as


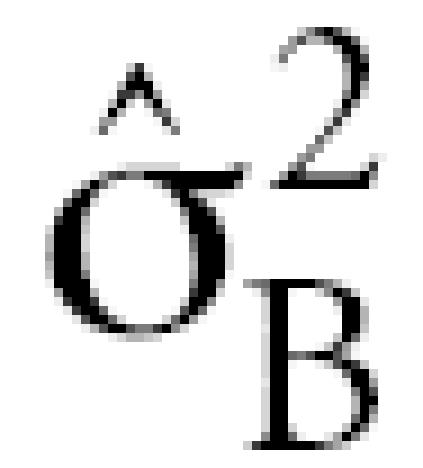


and,


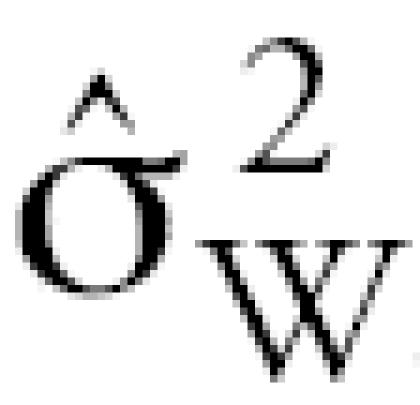


, respectively. We used


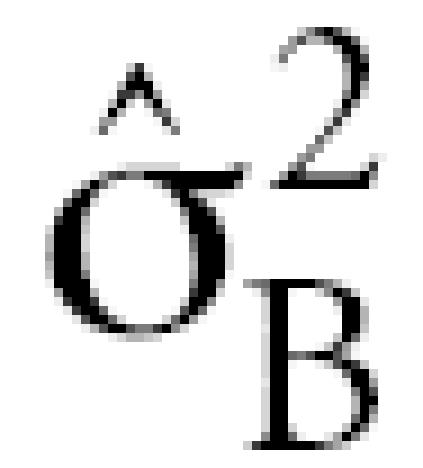


and


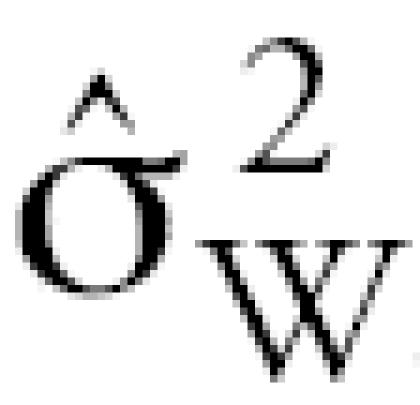


to estimate the intraclass correlation coefficient


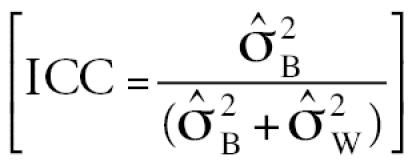


and the variance ratio


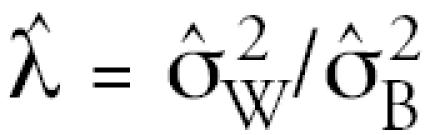


. The intra-class correlation coefficient (ICC) represents the estimated correlation between the *j*th and *j’*th observations on the *i*th subject and is often used as an index of reliability of a set of measurements (larger is better). The estimated variance ratio


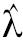


is a measure of the attenuation bias (smaller is better) when using a surrogate for true exposure (albumin adduct levels in this context) to predict an exposure–disease relationship (Lin et al. 2005). The SAS standard package for Windows version 8.2 (SAS Institute Inc., Cary, NC, USA) and S-PLUS 6.2 (Insightful Corp., Seattle, WA, USA) were used for statistical analyses, and the level of significance of all tests was 0.05.

## Results

### Assay precision and summary statistics

The CVs of the assays of the three albumin adducts were 0.358, 0.129, and 0.061 for BO-Alb, 1,2-BQ-Alb, and 1,4-BQ-Alb, respectively.

The distributions of population characteristics and exposure categories are summarized in Table 1. The following median values of individual GM air concentrations of benzene were estimated within 3 months of phlebotomy: 0.004 ppm for control workers (predicted from levels of urinary benzene, *n* = 140), 0.460 ppm for workers exposed to < 1 ppm benzene (*n* = 70), 2.07 ppm for workers exposed to 1 to < 10 ppm benzene (*n* = 149), and 19.1 ppm for workers exposed to ≥ 10 ppm benzene (*n* = 31). Differences were generally small in the distributions of BMI, sex, and alcohol use across exposure categories.

Table 2 shows the distributions of albumin adducts stratified by blood-collection medium and exposure category. The effect of the blood-collection medium was striking, particularly for 1,2-BQ-Alb and 1,4-BQ-Alb, with adduct levels decreasing in the order plasma EDTA >> plasma heparin > serum. Although the effect of the blood-collection medium was smaller for BO-Alb, significant differences in BO-Alb levels were nonetheless observed between collection media in two of the four exposure categories.

### Exposure–adduct relationships

Figure 2 shows log-scale scatter plots with loess trends for albumin adducts in relation to benzene exposure, after adjustment for blood-collection medium, age, BMI, and smoking status. Interestingly, all three adducts displayed nonlinear (hockey-stick–shaped) exposure–adduct relationships with inflection points in those exposed to benzene at approximately 0.5–5 ppm. The curves shown at benzene concentrations < 1 ppm represent the rather small contributions of benzene-derived adducts to adducts arising from unknown dietary and endogenous sources. Above 1 ppm, the contributions of benzene exposure to albumin adducts become apparent.

Given the nonlinear relationships between adduct levels and benzene exposure shown in Figure 2, we fit separate linear models of adducts levels for subjects exposed to < 1 ppm and ≥ 1 ppm. These models are summarized in Tables 3–5 for BO-Alb, 1,2-BQ-Alb, and 1,4-BQ-Alb, respectively. To compare among adducts, we retained smoking status, age, and BMI in final models even if they were not statistically significant. Six outliers of 1,2-BQ-Alb among individuals exposed to < 1 ppm (shown in Figure 2B), were excluded from multivariate regression analyses based on the generalized extreme-Studentized-deviation-many-outlier method (Rosner 1983).

Results of multivariate models showed that benzene exposure was a much stronger predictor of adduct levels among workers exposed to ≥ 1 ppm; indeed, only 1,4-BQ-Alb was significantly associated with benzene exposure < 1 ppm (regression coefficient β = 0.030, *p* = 0.010; Table 5). Among workers exposed to ≥ 1 ppm benzene, the regression coefficient of (logged) benzene exposure was much greater for BO-Alb (β = 0.668) than for 1,2-BQ-Alb (β = 0.393) or 1,4-BQ-Alb (β = 0.391).

As expected from summary analyses shown in Table 2, the blood-collection medium was an important predictor of levels of albumin adducts, particularly for 1,2-BQ-Alb and 1,4-BQ-Alb, where plasma collected in EDTA showed much higher adduct levels than serum (*p* < 0.001). In contrast, the effect of the blood-collection medium was small for BO-Alb, where it was significant only among workers exposed to < 1 ppm benzene. Due to the large effects of the blood-collection medium, parallel multiple regression models were performed with albumin adducts determined in serum only (*n* = 300). These analyses produced final models that were essentially the same as those shown in Tables 3–5 (data not shown).

Effects of other covariates differed among the albumin adducts and between exposure categories. For BO-Alb (Table 4), smokers exposed to ≥ 1 ppm benzene had marginally higher adduct levels than nonsmokers (*p* = 0.048), after adjustment for other covariates. The effects of cigarette smoking were much more pronounced for adducts of the benzoquinones in both low (< 1 ppm: *p* = 0.024 for 1,2-BQ-Alb and *p* < 0.001 for 1,4-BQ-Alb) and high (≥ 1 ppm: *p* = 0.007 for 1,2-BQ-Alb and *p* = 0.003 for 1,4-BQ-Alb) exposure categories (Tables 4, 5). Age and BMI tended to be negatively associated with the levels of all three albumin adducts (Table 3–5). However, the effect of age was significant only for 1,2-BQ-Alb among low-exposed subjects (*p* = 0.018) and was marginally significant for 1,4-BQ-Alb among high-exposed subjects (*p* = 0.078); the effect of BMI was significant only for 1,4-BQ-Alb for subjects exposed to ≥ 1 ppm benzene (*p* = 0.014).

Estimated between-person and within-person variance components, ICCs, and variance ratios are shown in Table 6, based on application of Equation 1 to adduct levels from 28 exposed workers with two blood specimens (median benzene concentration = 1.47 ppm; range: 0.353–42.9 ppm). The estimated between-person variance component (


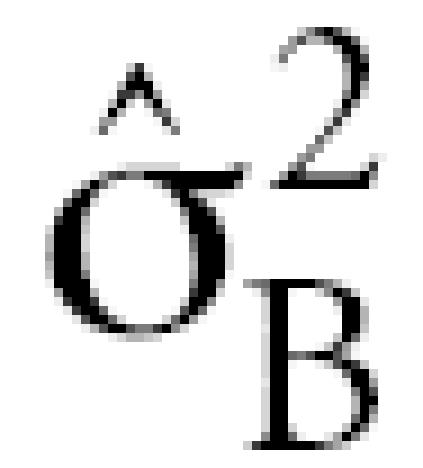


) increased in the following order: 1,2-BQ-Alb (0.044) < 1,4-BQ-Alb (0.521) < BO-Alb (1.59), whereas the estimated within-person variance component


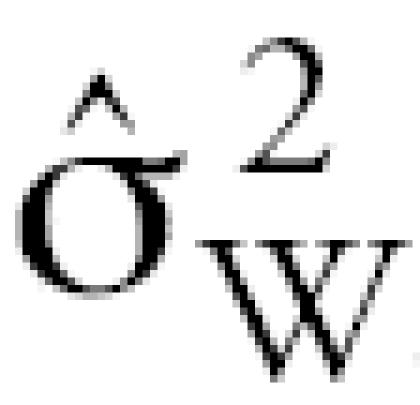


increased in the opposite order: BO-Alb (0.175) < 1,4-BQ-Alb (0.319) < 1,2-BQ-Alb (0.503). The values of the ICCs were 0.901, 0.080, and 0.620 for BO-Alb, 1,2-BQ-Alb, and 1,4-BQ-Alb, respectively, and the estimated variance ratios (


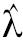


) were 0.110, 11.4, and 0.612, respectively (Table 6).

## Discussion

In this study of 390 Chinese workers, we observed that benzene exposures were associated with increased production of albumin adducts of BO, 1,4-BQ and 1,2-BQ. These findings confirm earlier associations between levels of BO-Alb and 1,4-BQ-Alb and benzene exposures in two other populations of Chinese workers (Rappaport et al. 2002a; Yeowell-O’Connell et al. 2001) and show that levels of 1,2-BQ-Alb, which had not been reported heretofore in humans, were also associated with benzene exposure at ≥ 1 ppm.

The shapes of exposure–adduct relationships in persons exposed to low levels of benzene from environmental air have not been reported previously. In the present study, we modeled adduct concentrations over about 5 orders of magnitude of benzene exposures (range, < 0.01–54.5 ppm), using benzene concentrations that had been predicted for control subjects from measurements of urinary benzene (Kim et al. 2006). The results, shown in Figure 2, point to hockey-stick-shaped relationships in log-scale between each of the three adducts and benzene exposure. The inflection points, which ranged from 0.5 to 5 ppm, represent air concentrations at which benzene contributed marginally to the pools of background adducts. Based on the curves in Figure 2, it appears that 1,4-BQ-Alb was the most responsive to benzene exposure, with an inflection point of about 0.5 ppm, followed by BO-Alb (~ 1–3 ppm) and 1,2-BQ-Alb (~ 5 ppm). Interestingly, the inflection points for 1,4-BQ-Alb and 1,2-BQ-Alb are comparable to those observed for urinary levels of hydroquinone and catechol (their respective precursors) in this same population of workers (Kim et al. 2006).

Our results also indicate that none of the three albumin adducts would be useful biomarkers of benzene exposure in ambient populations, where air concentrations rarely exceed 0.1 ppm, or in working populations where exposures are consistently maintained at < 1 ppm. Indeed, among workers exposed to air concentrations < 1 ppm, only 1,4-BQ-Alb showed a significant effect of benzene exposure (Figure 2, Table 5), and this reflects exposures between 0.1 and 1 ppm. When we fit the same regression model to levels of 1,4-BQ-Alb for workers exposed to ≤ 0.1 ppm benzene, the coefficient (± SE) for benzene exposure decreased from 0.030 (± 0.011) to 0.002 (± 0.032), with no hint of statistical significance (*p* = 0.940).

**Figure 1:**
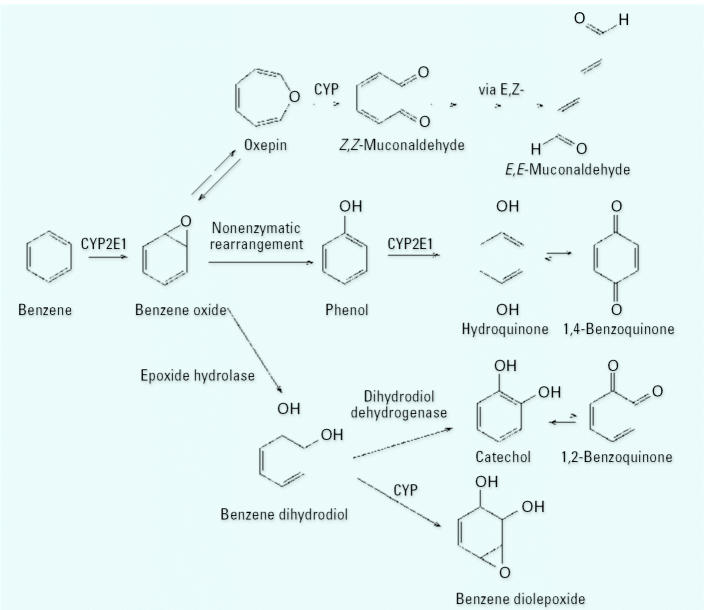
Pathways of benzene metabolism leading to reactive electrophilic species. CYP, cytochrome P450.

**Figure 2:**
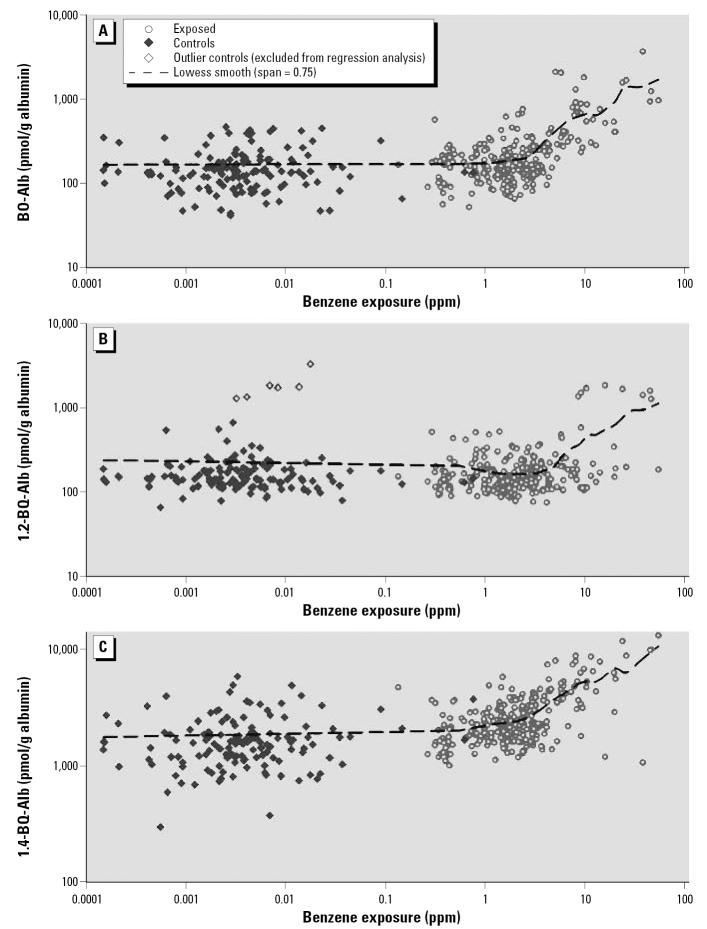
Levels of BO-Alb (*A*), 1,2-BQ-Alb (*B*), and 1,4-BQ-Alb (*C*) at increasing air concentrations of benzene, after adjusting for blood-collection medium, age, BMI, and cigarette smoking. Dashed lines indicate a loess smooth function of benzene exposure derived from a generalized additive model, with adjustment for blood-collection medium, age, BMI, and cigarette smoking. In (*B*), note the six outliers, which were excluded from multiple regression analyses.

**Figure 3:**
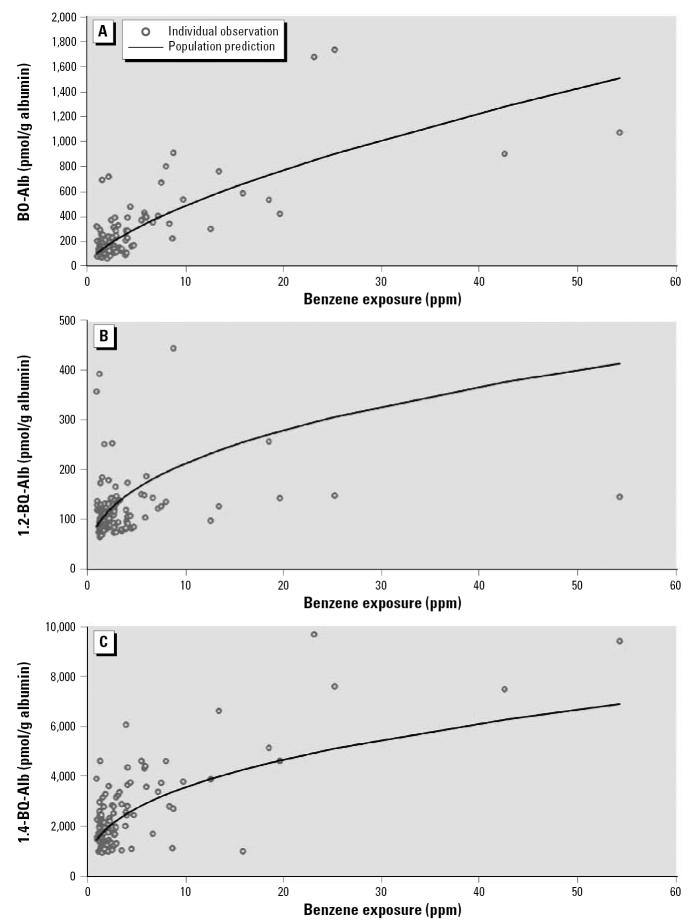
Predicted natural-scale relationships between levels of BO-Alb (*A*), 1,2-BQ-Alb (*B*), and 1,4-BQ-Alb (*C*). Data points represent adduct levels derived from serum from nonsmoking subjects of average weight and BMI, with benzene exposure of ≥ 1 ppm; curves show adduct levels predicted from multivariate linear models shown in Tables 3–5.

Because all exposure–adduct relationships were reasonably modeled by simple linear models (in log-scale) > 1 ppm (Figure 2), we fit separate multiple regression models to workers exposed to benzene either < 1 ppm or ≥ 1 ppm. This allowed us to compare effects of benzene exposure on adduct production after adjusting for the blood-collection medium, age, BMI, and smoking (Tables 3–5). For workers exposed to benzene ≥ 1 ppm, the log-scale regression coefficients for benzene exposure and their upper 95% confidence limits (UCL) were all < 1 [i.e., BO-Alb: β = 0.668 (UCL = 0.770); 1,2-BQ-Alb: β = 0.393 (UCL = 0.495); and 1,4-BQ-Alb: β = 0.391 (UCL = 0.469)]. This indicates that the natural-scale relationships between adduct levels and benzene exposures were concave downward in all cases, as observed previously for BO-Alb and 1,4-BQ-Alb (Rappaport et al. 2002a, 2002b). Furthermore, the magnitude of each adjusted coefficient for benzene exposure in Tables 3–5 indicates the degree of concavity of the respective exposure–adduct relationship in natural scale; that is, the smaller the log-scale coefficient, the greater the concavity in natural scale. This is illustrated in Figure 3, which shows predicted natural-scale relationships corresponding to the coefficients estimated from the multiple linear regression models. These curves represent adduct levels in serum of nonsmoking workers of average age and average BMI with GM benzene exposures of ≥ 1 ppm. The relationships for the two benzoquinone adducts show greater concavity than that of BO-Alb. If these concave-downward relationships are the result of saturable metabolism of benzene, as suggested previously in studies of animals (Medinsky et al. 1989; Sabourin et al. 1988) and of humans (Rappaport et al. 2002a, 2002b), then our results indicate that the saturable effects are greater for metabolism to the benzoquinones than for metabolism to BO (Figure 1).

We found that levels of 1,2-BQ-Alb and 1,4-BQ-Alb were much higher in plasma containing EDTA than in either serum or plasma containing heparin (Table 2). While we do not know the underlying reason for this result, it probably explains the large difference in 1,4-BQ-Alb levels, which had been observed previously in two studies of benzene-exposed workers (Rappaport et al. 2005). In those studies, plasma containing EDTA contained much higher levels of 1,4-BQ-Alb than plasma containing citrate, and the difference disappeared when adduct levels were adjusted for concurrent controls. Because EDTA is a well-known chelating agent, it is worth speculating that chelation of iron would stabilize benzoquinone adducts, possibly by inhibiting Fenton chemistry. Additional work should be conducted to determine why the blood-collection medium would have such a large effect upon levels of albumin adducts of the benzoquinones.

Regarding effects of smoking, age, and BMI, results of multiple regression models varied among the three types of adducts and between exposure categories (Tables 3–5). Among subjects exposed to benzene ≥ 1 ppm, smoking was positively associated with levels of BO-Alb (β = 0.209), 1,2-BQ-Alb (β = 0.286), and 1,4-BQ-Alb (β = 0.242), indicating that GM adduct levels were between 23% (i.e., e^0.209^) and 33% (i.e., e^0.286^) higher in smokers than in nonsmokers. For subjects exposed to < 1 ppm of benzene, smokers had 15% more 1,2-BQ-Alb (β = 0.139) and 58% more 1,4-BQ-Alb (β = 0.456) than nonsmokers, whereas levels of BO-Alb were virtually unaffected by smoking (β = 0.016). These results point to the likely contributions of hydroquinone and catechol (precursors of 1,4-BQ and 1,2-BQ, respectively) in cigarette smoke (Kim et al. 2006).

Adducts of the benzoquinones decreased with age at about 0.8%/year of life for 1,2-BQ-Alb in low-exposed workers (β = −0.008) and 1,4-BQ-Alb in high-exposed workers (β = −0.008). In a previous study of Chinese workers exposed over a similar range of air concentrations, 1,4-BQ-Alb levels decreased by 1.9%/year of life among both exposed and control workers (Rappaport et al. 2002a).

Workers were also exposed to toluene at a median concentration of 3.36 ppm (range, < 0.3–80.9 ppm) (data not shown). Because toluene competes with benzene for cytochrome P450 2E1 metabolism, we anticipated that levels of albumin adducts would decrease with toluene exposure among workers exposed to benzene at ≥ 1 ppm. However, when toluene exposure was added to models of the three albumin adducts, the effects were not significant and regression coefficients for benzene exposure were only marginally reduced (3–4%).

Because 28 exposed subjects had two blood specimens (collected about 16 months apart), it was possible to estimate within-person and between-person variance components for the (logged) levels of albumin adducts, after adjustment for blood-collection media. The estimated within-person variance component (


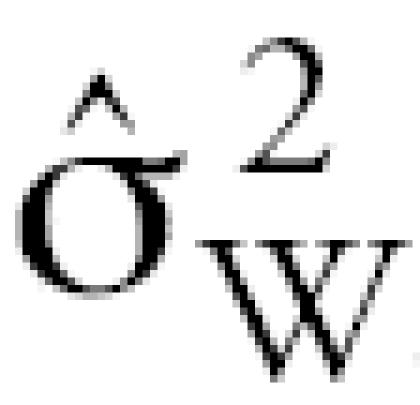


) increased in the order BO-Alb < 1,4-BQ-Alb < 1,2-BQ-Alb, whereas the estimated between-person variance component (


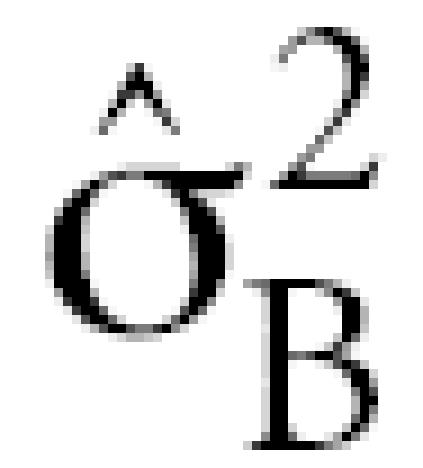


) showed the opposite behavior. Because


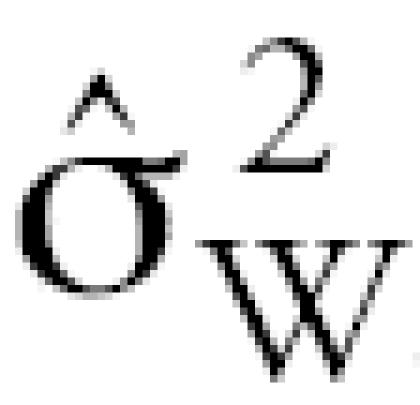


tends to decrease with increasing residence time of a biomarker (Lin et al. 2005), this finding is consistent with a previous report that BO-Alb was chemically stable in humans, turning over with albumin (half life = 21 days), whereas 1,4-BQ-Alb was marginally unstable (half life = 13.5 days) (Rappaport et al. 2002a); the finding also suggests that 1,2-BQ-Alb is very unstable in humans.

This disparity in values of


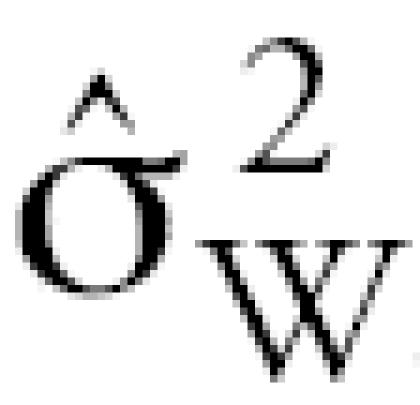


and


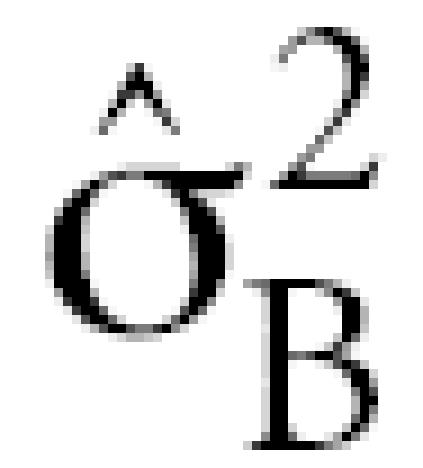


for the three albumin adducts influenced the corresponding values of the


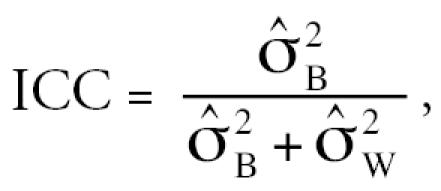


a measure of reliability (larger is better), and of the variance ratio


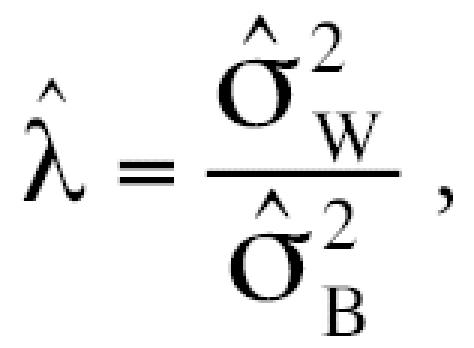


a measure of the biasing potential of the biomarker as a surrogate for exposure (smaller is better) (Lin et al. 2005). Because BO-Alb had the largest ICC (0.901) and the smallest


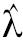


(0.110), followed by 1,4-BQ-Alb (ICC = 0.620,


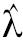


= 0.612) and 1,2-BQ-Alb (ICC = 0.080,


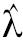


= 11.4), BO-Alb should be the most reliable and least biasing biomarker of occupational exposure to benzene of the three adducts measured in our study.

The estimated within-person variance components for BO-Alb (


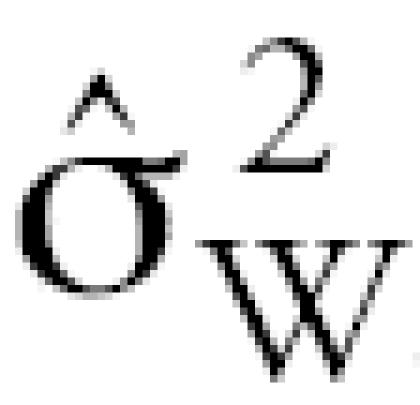


= 0.175) and 1,4-BQ-Alb (


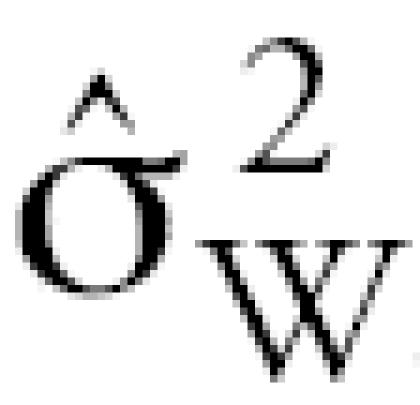


= 0.319) in the present study were larger than those estimated previously (


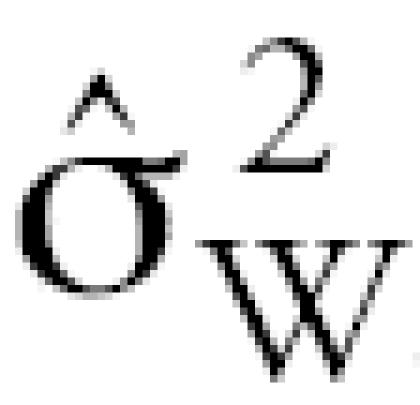


= 0.079 and 0.044, respectively) from 11 benzene exposed workers in China who provided blood samples on three consecutive Mondays (Rappaport et al. 2002a). The larger estimates of


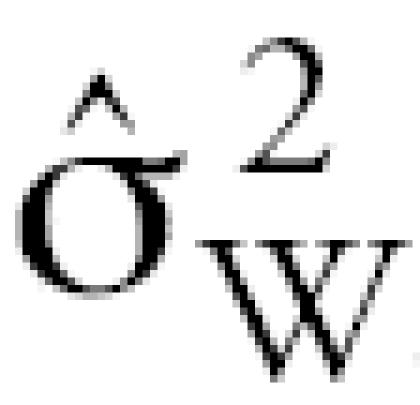


in the present study were probably influenced by the much larger interval between blood samples (about 16 months) (Lin et al. 2005).

In conclusion, the present study confirms and extends previous observations of concave downward relationships between albumin adducts of biologically reactive benzene metabolites and benzene exposure (Figure 3) (Rappaport et al. 2005). We attribute this nonlinear behavior to saturable metabolic processes involving the production of BO, 1,2-BQ, and 1,4-BQ in humans (Figure 1). Because levels of these reactive and hematotoxic (at least in the case of 1,4-BQ) benzene metabolites were less than proportional to benzene exposure at air concentrations in the range of 1–10 ppm (Figure 3), risk assessments that were based largely upon linear fits of leukemia mortality among workers exposed to hundreds of parts per million of benzene could well underestimate risks from benzene metabolites in persons exposed at lower (non-saturating) air concentrations (Rappaport et al. 2005). In addition, this study highlights the importance of nonoccupational sources that also contribute to benzene-related adducts. These background adducts limit the usefulness of albumin adducts as biomarkers of benzene exposure below about 1 ppm. On the other hand, given the established causal association between human leukemia and benzene exposure, further investigation of these adducts in low-exposed subjects may help explain unknown causes for leukemia in the general population (Lin et al. 2006; McDonald et al. 1994, 2001; Smith 1996).

## Figures and Tables

**Table 1:** Summary statistics of population demographic characteristics [median (range) or *n* (%)].

	Controls (*n* = 140)^a^	< 1 ppm (*n* = 70)	1 to < 10 ppm (*n* = 149)	≥ 10 ppm (*n* = 31)	*p-*Value^b^
Age (years)	28 (18–51)	28 (19–46)	27 (18–49)	36 (21–52)	0.02
BMI (kg/m^2^)	21.6 (16.0–38.5)	22.3 (15.4–30.1)	22.0 (15.4–32.3)	21.3 (17.7–28.3)	0.89
Sex [*n* (%)]					0.30
Male	52 (37)	30 (43)	45 (30)	11 (35)	
Female	88 (63)	40 (57)	104 (70)	20 (65)	
Current alcohol use [*n* (%)]					0.41
Yes	43 (31)	20 (29)	35 (23)	11 (35)	
No	97 (69)	50 (71)	114 (77)	20 (65)	
Current smoker [*n* (%)]					0.19
Yes	39 (28)	17 (24)	26 (17)	8 (26)	
No	101 (72)	53 (76)	123 (83)	23 (74)	
Urinary benzene (μg/L)	0.120 (0.007–10.1)	7.89 (0.667–72.5)	23.8 (0.50–1,400)	359 (5.20–4,210)	< 0.001
Airborne benzene (ppm)	0.004 (< 0.01–0.53)^c^	0.46 (0.26–1.00)	2.07 (1.02–9.87)	19.1 (10.0–54.5)	< 0.001

a One missing datum for urinary (airborne) benzene (*n* = 139), and two missing data for BMI (*n* = 138).

b Wilcoxon rank-sum test or chi-square test between smokers and nonsmokers.

c Estimated from urinary benzene concentrations.

**Table 2:** Albumin adducts of benzene stratified by exposure category and blood-collection medium [median (range; *n*)].

	Controls^a^	< 1 ppm	1 to < 10 ppm^b^	≥ 10 ppm
BO-Alb (pmol/g)				
Plasma EDTA	100 (70.9–155; *n* = 11)	172 (109–208; *n* = 10)	188 (134–261; *n* = 9)	—c
Plasma heparin	99.4 (57.4–241; *n* = 19)	208 (208–208; *n* = 1)^b^	350 (106–698; *n* = 10)	528 (121–3,060; *n* = 19)
Serum	152 (66.3–486; *n* = 109)	163 (54.4–573; *n* = 59)	165 (68.8–917; *n* = 129)	838 (307–3,920; *n* = 12)
*p*-Value	< 0.001	0.61	0.04	0.24
1,2-BQ-Alb (pmol/g)				
Plasma EDTA	1,240 (859–1,810; *n* = 11)	1,670 (1,230–2,360; *n* = 10)	1,030 (530–1,460; *n* = 9)	—c
Plasma heparin	204 (72.2–737; *n* = 19)	169 (169–169; *n* = 1)^d^	206 (100–394; *n* = 10)	222 (147–539; *n* = 19)
Serum	125 (71.3–2,480; *n* = 109)	106 (69.4–397; *n* = 59)	110 (66.4–1,280; *n* = 129)	204 (98.9–1,420; *n* = 12)
*p*-Value	< 0.001	< 0.001	< 0.001	0.94
1,4-BQ-Alb (pmol/g)				
Plasma EDTA	7,080 (5,450–18,600; *n* = 10)	6,990 (5,520–9,640; *n* = 10)	7,030 (4,530–30,100; *n* = 9)	—c
Plasma heparin	1,520 (466–3,210; *n* = 19)	5,590 (5,590–5,590; *n* = 1)^d^	3,990 (1,870–6,520; *n* = 10)	6,020 (2,310–13,300; *n* = 19)
Serum	1,340 (420–12,600; *n* = 109)	1,710 (905–5,270; *n* = 59)	2,100 (953–6,100; *n* = 129)	5,900 (1,020–11,300; *n* = 12)
*p*-Value	< 0.001	< 0.001	< 0.001	0.63

Nonparametric Kruskal-Wallis tests were used to compare adduct levels among three blood-collection media within each exposure category.

a One missing datum for BO-Alb and 1,2-BQ-Alb (*n* = 139), and two missing data for 1,4-BQ-Alb (*n* = 138).

b One missing datum for all albumin adducts (*n* = 148).

c No data available.

d Excluded from the analysis because there was only one observation.

**Table 3:** Multivariate linear regression models for BO-Alb.^a^,^b^

	< 1 ppm (*n* = 206)^c^		≥ 1 ppm (*n* = 179)^c^	
	β (SE)	*p-*Value	β (SE)	*p-*Value
Intercept	5.18 (0.220)	< 0.001	5.21 (0.316)	< 0.001
Benzene (ppm)^a^	0.002 (0.013)	0.884	0.668 (0.052)	< 0.001
Blood-collection medium		< 0.001		0.149
Plasma EDTA	−0.246 (0.110)	0.027	0.133 (0.187)	0.478
Plasma heparin	−0.407 (0.114)	< 0.001	−0.250 (0.138)	0.071
Serum	Reference		Reference	
Age (years)	0.002 (0.004)	0.611	−0.003 (0.005)	0.538
BMI (kg/m^2^)	−0.007 (0.010)	0.470	−0.020 (0.013)	0.128
Smoke cigarettes	0.016 (0.076)	0.834	0.209 (0.105)	0.048
*R*^2^ (adjusted *R*^2^)	0.08 (0.05)		0.57 (0.56)	

a Log-transformed.

b For the sake of comparison, nonsignificant covariates were also retained in the model.

c Because of missing adduct determinations, four observations were excluded from the group exposed to < 1 ppm benzene and one observation was excluded from the group exposed to ≥ 1ppm.

**Table 4:** Multivariate linear regression models for 1,2-BQ-Alb.^a^,^b^

	< 1 ppm (*n* = 200)^c^		≥ 1 ppm (*n* = 179)^c^	
	β (SE)	*p-*Value	β (SE)	*p-*Value
Intercept	5.18 (0.181)	< 0.001	4.31 (0.318)	< 0.001
Benzene (ppm)^a^	−0.001 (0.010)	0.889	0.393 (0.052)	< 0.001
Blood-collection media		< 0.001		< 0.001
Plasma EDTA	2.41 (0.088)	< 0.001	2.14 (0.188)	< 0.001
Plasma heparin	0.405 (0.091)	< 0.001	−0.162 (0.139)	0.244
Serum	Reference		Reference	
Age (years)	−0.008 (0.003)	0.018	0.000 (0.005)	0.999
BMI (kg/m^2^)	−0.007 (0.008)	0.368	0.007 (0.013)	0.607
Smoke cigarettes	0.139 (0.061)	0.024	0.286 (0.105)	0.007
*R*^2^ (adjusted *R*^2^)	0.81 (0.80)		0.52 (0.50)	

a Log-transformed.

b For the sake of comparison, nonsignificant covariates were also retained in the model.

c Because of missing adduct determinations, four observations were excluded from the group exposed to < 1 ppm benzene and one observation was excluded from the group exposed to ≥ 1ppm. Six outliers were excluded from the group exposed to < 1 ppm.

**Table 5:** Multivariate linear regression models for 1,4-BQ-Alb.^a^,^b^

	< 1 ppm (*n* = 205)^c^		≥ 1 ppm (*n* = 179)^c^	
	β (SE)	*p-*Value	β (SE)	*p-*Value
Intercept	7.78 (0.196)	< 0.001	8.07 (0.245)	< 0.001
Benzene (ppm)^a^	0.030 (0.011)	0.010	0.391 (0.040)	< 0.001
Blood-collection media		< 0.001		< 0.001
Plasma EDTA	1.55 (0.100)	< 0.001	1.32 (0.145)	< 0.001
Plasma heparin	−0.052 (0.101)	0.608	0.230 (0.107)	0.033
Serum	Reference		Reference	
Age (years)	−0.005 (0.004)	0.144	−0.008 (0.004)	0.078
BMI (kg/m^2^)	−0.013 (0.008)	0.134	−0.025 (0.010)	0.014
Smoke cigarettes	0.456 (0.068)	< 0.001	0.242 (0.081)	0.003
*R*^2^ (adjusted *R*^2^)	0.62 (0.61)		0.60 (0.59)	

a Log-transformed.

b For the sake of comparison, nonsignificant covariates were also retained in the model.

c Because of missing adduct determinations, five observations were excluded from the group exposed to < 1 ppm benzene and one observation was excluded from the group exposed to ≥ 1 ppm.

**Table 6:** Estimated variance components of (log-transformed) levels of albumin adducts.^a^

	BO-Alb	1,2-BQ-Alb	1,4-BQ-Alb
Between-person variance ( 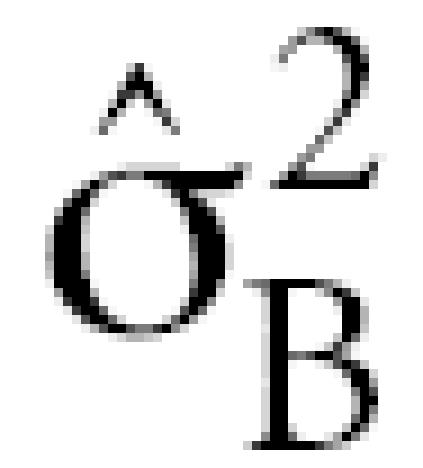 )	1.59	0.044	0.521
Within-person variance ( 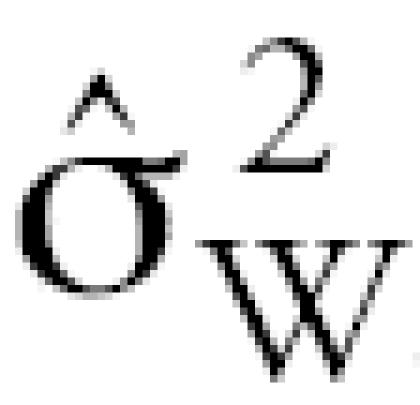 )	0.175	0.503	0.319
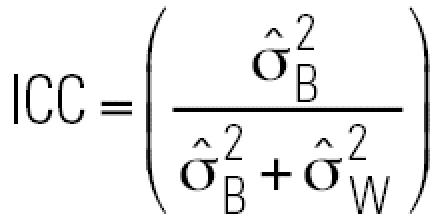	0.901	0.080	0.620
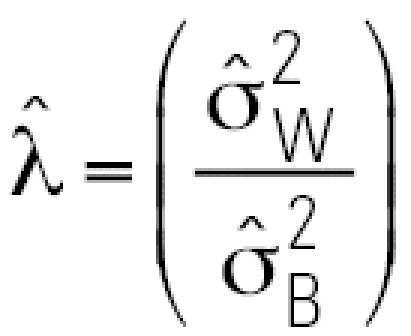	0.110	11.4	0.612

a Based on a subgroup of 28 benzene-exposed workers; adjusted for blood-collection medium.
